# Endocrine Challenges in Patients with Continuous-Flow Left Ventricular Assist Devices

**DOI:** 10.3390/nu13030861

**Published:** 2021-03-05

**Authors:** Gennaro Martucci, Federico Pappalardo, Harikesh Subramanian, Giulia Ingoglia, Elena Conoscenti, Antonio Arcadipane

**Affiliations:** 1 Department of Anesthesia and Intensive Care, IRCCS-ISMETT (Istituto Mediterraneo per i Trapianti e Terapie ad Alta Specializzazione), 90133 Palermo, Italy; fpappalardo@ismett.edu (F.P.); aarcadipane@ismett.edu (A.A.); 2 Department of Anesthesiology and Perioperative Medicine, University of Pittsburgh Medical Center, Pittsburgh, PA 15201, USA; subramanianh4@upmc.edu; 3 Section of Anesthesia Analgesia Intensive Care and Emergency, Department of Surgical, Oncological and Oral Science, University of Palermo, 90133 Palermo, Italy; ingogiulia@gmail.com; 4 Infectious Disease and Infection Control Service, IRCCS-ISMETT (Istituto Mediterraneo per i Trapianti e Terapie ad Alta Specializzazione), 90133 Palermo, Italy; econoscenti@ismett.edu

**Keywords:** ventricular assist devices, VAD, heart failure, TSH, infections, anemia, erythropoietin

## Abstract

Heart failure (HF) remains a leading cause of morbidity, hospitalization, and mortality worldwide. Advancement of mechanical circulatory support technology has led to the use of continuous-flow left ventricular assist devices (LVADs), reducing hospitalizations, and improving quality of life and outcomes in advanced HF. Recent studies have highlighted how metabolic and endocrine dysfunction may be a consequence of, or associated with, HF, and may represent a novel (still neglected) therapeutic target in the treatment of HF. On the other hand, it is not clear whether LVAD support, may impact the outcome by also improving organ perfusion as well as improving the neuro-hormonal state of the patients, reducing the endocrine dysfunction. Moreover, endocrine function is likely a major determinant of human homeostasis, and is a key issue in the recovery from critical illness. Care of the endocrine function may contribute to improving cardiac contractility, immune function, as well as infection control, and rehabilitation during and after a LVAD placement. In this review, data on endocrine challenges in patients carrying an LVAD are gathered to highlight pathophysiological states relevant to this setting of patients, and to summarize the current therapeutic suggestions in the treatment of thyroid dysfunction, and vitamin D, erythropoietin and testosterone administration.

## 1. Introduction

Chronic heart failure (CHF) remains a leading cause of morbidity, hospitalization, and mortality worldwide [1]. The advancements in technology and clinical management have promoted an increased application of continuous-flow left ventricular assist devices (LVADs) as a long-term therapy, either as a bridge-to-transplantation or, increasingly, “bridge to destination” [2]. In this picture, LVADs are able to reduce hospitalizations, and improve the quality of life and mid-term outcomes of advanced HF patients [3].

Recent studies have highlighted how metabolic and endocrine dysfunction are a strong morbid consequence of, or accompanied in HF, and may represent a novel (probably still neglected) therapeutic target in the treatment of HF [4]. On the other hand, LVADs that impact the outcome by improving the peripheral and end-organ perfusion, may also improve the neurohormonal state of patients, reducing the endocrine dysfunction [5,6,7,8]. However, endocrine function is likely a major determinant of human homeostasis, and is a key issue in the recovery from critical illness [9]. The active care of endocrine function may be relevant in improving immune function, cardiac homeostasis, and rehabilitation during and after a LVAD placement.

On the topic of endocrine dysfunction in LVAD, intuitively a great topic of speculation is adrenal function and insulin metabolism. HF patients are likely chronically adrenal-depleted and, the effect of adrenal deficiency (relative or absolute) is well described in the literature in terms of hemodynamic impairment, immunosuppression, perioperative infections, as well as wound and surgical healing [10,11]. Regarding insulin, diabetes is a major comorbidity in the HF setting, and the need for adequate balancing and also increase in the therapy to avoid fluctuations of glycemia in the perioperative period are a major issue [12]. Both these topics are not covered since they would require a manuscript for each part.

In this review, data on endocrine challenges in patients affected with HF and carrying an LVAD are gathered to highlight pathophysiological states relevant to this setting of patients, and to group the current therapeutic suggestions in the treatment of thyroid dysfunction, and vitamin D, erythropoietin, and testosterone administration. The hemodynamic platform provided by the LVAD maintains full circulatory support and reverses low cardiac output and systemic congestion; yet, the maladaptive response triggered by chronic heart failure is not shortly reversed. Referring to reversal of nutritional and endocrine issues, these require full post-surgical recovery and restoration of stable cardiopulmonary reserve and end-organ function. Indeed, the biological challenge is kept at a “low tune” by the physiological characteristics of LVAD support (continuous flow, GI bleeding, bioartificial surfaces, chronic infection), calling for an extensive application of therapies transferred from the heart failure arena.

Thyroid dysfunction (TD) is a known cause of HF, but several causes favor the occurrence of TD before and after LVAD placement: altered thyroid gland perfusion, neurohormonal blockade, and drugs with a tropism for the thyroid, such as amiodarone. Therefore, thyroid-stimulating-hormone (TSH) increase is a common finding in LVAD patients in the framework of the euthyroid sick syndrome when there is not a clear hypothyroidism.

Vitamin D is a major determinant of prognosis in critically ill patients. It is a hormone with pleiotropic functions implicated in many homeostatic functions. Several data have highlighted how vitamin D deficiency is a negative condition associated with poor outcome in cardiac surgery and HF patients. Despite the fact that the effect on mortality is not well defined with randomized controlled trials, its effect in increasing innate immunity, and reducing chronic inflammation render vitamin D a suitable supplementary drug for LVAD patients.

Erythropoietin is widely used in patients with end-stage chronic renal failure on dialysis since they are considered deprived of the hormonal kidney function. In HF its use is still underconsidered, but in the context of LVAD, with chronic anemia, potential for hemolysis, and need for chronic unloading its use have a strong potential plausibility.

Testosterone is a well-known anabolic hormone with a relevant role in vascular tone, and specific effects on endothelium and myocardial cells. Its use in critical care is still sporadic, but has all the potential to be a very important tool in rehabilitating VAD carriers.

## 2. Thyroid Dysfunction

Thyroid hormone (TH) signaling is a relevant component of the adaptive response of the myocardium to stress, and plays a critical role in regulating both heart rate and contractility of myocytes: hyperthyroidism is correlated with atrial arrhythmias, hypertension, and heart failure, and increases the risk of heart failure and mortality in cardiopathic patients [13]. As in the majority of hormones, TH is regulated by a number of pathways, and its production and endocrine release is impaired by the continuous flow determined by VADs.

The thyroid gland secretes T3 (triiodothyronine) and T4 (levothyroxine) under stimulation by TSH, the pituitary synthesis of which is downregulated with feedback loop mechanism by T3 and T4, and upregulated from hypothalamic synthesis of thyrotropin releasing hormone (TRH). The thyroid gland primarily secretes T4, which is then de-iodinated in its active form, T3, in the liver, kidney, and skeletal muscle.

### 2.1. Effects of Thyroid Hormones on Cardiac Function

The effect determined by TH on myocyte contractility is due to both genomic and non-genomic mechanisms. T3 binds thyroid hormones receptors (TRs) on the cellular membrane, promoting transcription processes involved in the synthesis of proteins responsible for cardiac contractility. For what concerns non-genomic activities, T3 can induce changes in the myocyte membrane, involving changes in sodium, potassium, and calcium ion channels. Both mechanisms lead to upregulation of sarcoplasmic reticulum calcium-activated ATPase.

**Figure 1:**
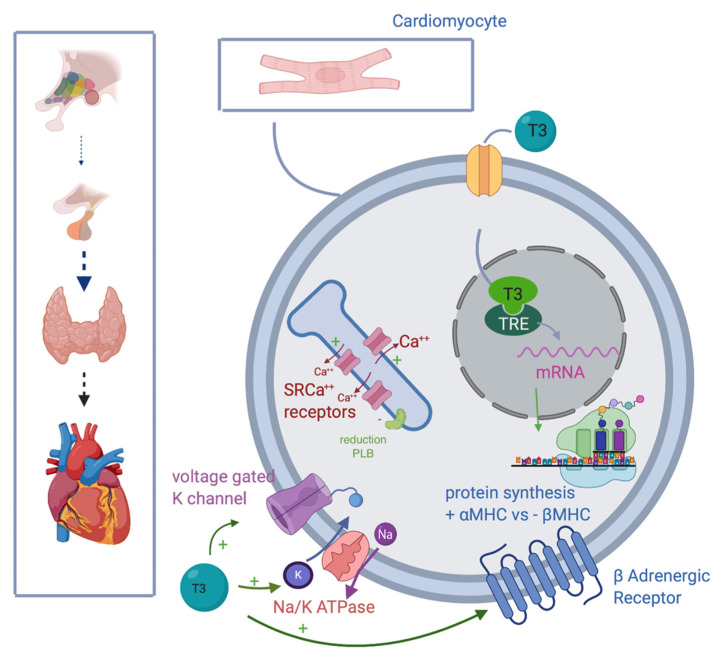
T3 improves myocytes and heart contractility, inducing intranuclear mRNA transcription, improving synthesis of fast αMHC, and reducing one of the slow βMHC; stimulating transmembrane expression of voltage-gated K channel, Na/K ATPase and βadrenergic receptors; upregulating SRCa^++^ rec, while inhibiting PLB. Abbreviations: T3, triiodothyronine; mRNA, messenger RNA; αMHC, α myosin heavy chain; Na/K ATPase, sodium-potassium adenosine triphosphatase; SRCa^++^ receptors, sarcoendoplasmic reticulum (SR) Ca(2+) receptors; PLB, phospholambane; TRE, thyroid hormones response element; βMHC, β myosin heavy chain.

In summary, TH acts on the cardiac function in the following ways (Figure 1):

- Promoting synthesis of the α isoforms of the myosin heavy chain (faster contractility), and reducing β isoform (slow contractility), enhancing systolic function.
- Upregulating β1 receptors, improving adrenergic responsiveness.
- Upregulating Na/K ATPase and voltage gating potassium channel [14].
- Inducing sarcoendoplasmic reticulum (SR) Ca(2^+^) receptors SERCA2a and down-regulation of phospholamban ATPase.
- Acting on the sinoatrial node, T3 can also improve heart rate.

On the other hand, transcription is inhibited in the absence of T3, inducing depression of myocardial function [13,14]. Moreover, acting on vascular smooth muscle cells (VSM), T3 decreases vascular resistance, improving arterial compliance, with a direct effect on VSM, and stimulating production of nitric oxide, with a 50% cardiac output increase [15]. Hypothyroidism is characterized by the opposite scenario: increased resistance and reduction of cardiac output and contractility. Moreover, heart failure can be related to altered conversion of T4 to T3.

### 2.2. Considerations after LVAD Implant and Potential Implications for Supplementation

VAD determines prolonged cardiac unloading, leading to molecular and structural modifications in heart cells. According to Ito et al., unloading is responsible for impaired Ca^++^ homeostasis, switch from α to β MHC (myocardial heavy chains) protein synthesis, and suppression of SERCA function. All of these parameters improve after T3 administration, and several studies have shown that treatment with T3 could hinder impaired Ca^++^ homeostasis and the shift from α to β MHC, improving cardiac contractility. Moreover, T3 is responsible for the restoration of cardiac activity contrasting PLB (phospholambane) phosphorylation suppression induced by VAD. ATPase function is also suppressed, and improves after treatment with T3 [16].

The benefits induced by T3 treatments are stressed in a case report described by Letsou in 2013 concerning the insurgence of thyrotoxicosis in a young patient affected with CHF on VAD. They observed the resolution of heart failure after an episode of thyrotoxicosis, and stressed the improvement in contractility function to the high level of TH. Once THs went back to their normal levels, the pathology seemed to be resolved [17]. In another study, Dipla et al. investigated the role of LVADs in improving myocyte contractility and their responsiveness to isoprenaline. Their findings highlighted the positive response of the myocytes, attributing the contractile improvements to changes in Ca^++^ homeostasis and SRCa ATPase activity. Furthermore, β responsiveness to isoprenaline was investigated in one study. Isoprenaline upregulated the expression of β receptors, already downregulated by the long-term exposure to sympathetic agonists in HF patients, thus allowing a reduction in inotropic drug administration. Moreover, changes in PLB homeostasis were hypothesized [18].

Afterload reduction and increasing of myocyte contractility are essential elements in the therapy of HF [18]. TH can change myocyte contractility, modifying Ca^++^ homeostasis, SRCa, ATPase, upregulating β receptors, and stimulating NO secretion from smooth muscle cells. They could conceivably represent a possible resource in CHF treatment. Or, at least, thyroid hormone homeostasis should be considered central in patients with CHF and VAD.

CHF pattern is characterized by an altered pathway of synthesis of myocyte heavy chains (MHC), and this has been linked to modification in thyroid hormones axis; more precisely to a different pathway of TR (thyroid receptors) as a stress response to adrenergic stimuli [19,20].

Current therapies for HF are based on optimizing cardiocirculatory hemodynamics without focusing on myocyte remodeling. Pantos et al. illustrated the role of T3 in restoring myocyte contractility, noticing that low T3, known as low T3 syndrome, is considered a risk factor for HF and increased mortality, while a spontaneous recovery of T3 level is evidence of a better prognosis. Moreover, TH has been used after heart transplantation in order to improve the prognosis. Since the principle use of T4 instead of T3 led to inconsistent results, the real benefits have to be confirmed [21]. In any event, given the complex correlations between different factors in VAD carriers, TH may not be considered merely a bystander and a signal of general improvement, but every minimal part of the homeostatic restoration should be considered during recovery [13,15].

On the other hand, hyperthyroidism is associated with tachyarrhythmias and pulmonary hypertension, both detrimental after VAD placement, even more when associated with chronic myocardial ischemia. Thus, it is important to monitor and maintain the correct plasma level of TH.

Nguyen et al. found that in their cohort of patients affected with thyroid impairment, an improvement of their metabolic status was observed after implantation of VAD [6]. These studies suggest the possibility that T3 therapy could improve patient response to VAD placement, sustaining cardiac contractility and reducing vascular resistance.

Thyroid function in LVAD carriers and with HF likely still needs to be elucidated, but currently a reasonable balance between risks and benefits impose that attention should be given to TH measurements, and supplementation should be started early in hypothyroidism, as well as in the case of normal free fractions of the hormones with high TSH levels. This approach is not derived from studies on LVAD patients or HF patients, but is a common value for critically ill patients. The relevant topic is already a constant and protocolized monitoring to detect early potential dysfunction.

## 3. Vitamin D Deficiency and Supplementation

Vitamin D is a pre-hormone that has a key function in bone health and calcium metabolism. Across the last decade, this molecule has seen renewed attention by the healthcare community in several fields, since its receptor has been found in almost all cells and tissues of the human body, and several endocrine paracrine and autocrine functions have been elucidated. Moreover, currently, at a population level, vitamin D deficiency is diffused worldwide in a pandemic fashion for reasons still unclear, but likely linked to changes in the consumption of food, life-style attitudes, obesity, ageing, and high prevalence of chronic cardiovascular and immunity-related diseases [22]. Lower vitamin D levels are associated with cardiac, pulmonary, and metabolic diseases, as well as with general morbidity and mortality. An important role for vitamin D is clearly defined for regulating immunity, either in modulation in the case of autoimmune diseases, or increasing the power of innate immunity for prevention and response to viral and bacterial infections [23,24,25]. This association is even more relevant in critically ill patients, where vitamin D can be considered a real pro-survival hormone [26]. But, despite much interesting data and a strong pathophysiological scenario, evidence from standard randomized controlled trials is still far from conclusive [27,28].

### 3.1. Effects on Cardiac Function

**Figure 2:**
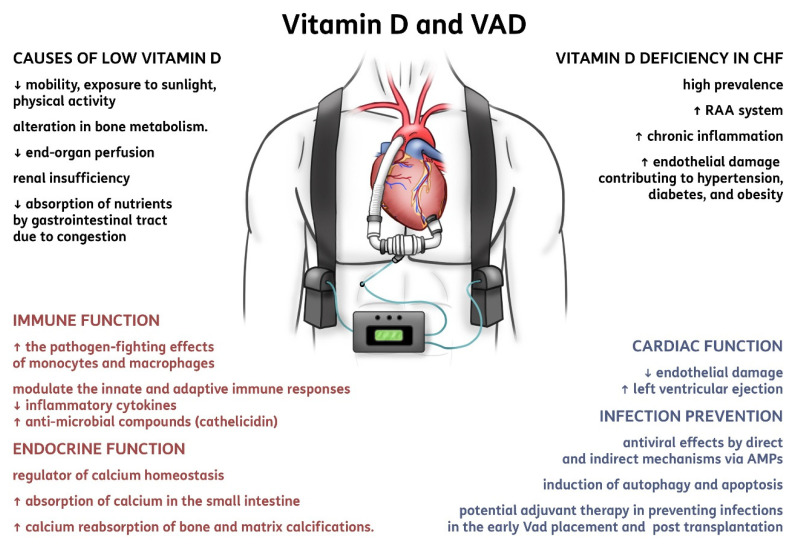
Summary of vitamin D implications in ventricular assist devices. Abbreviations: CHF, chronic heart failure; RAA system, renin-angiotensin-aldosterone system; VAD, ventricular assist device; AMPs, anti-microbial peptides.

In the cases of the heart dysfunction, vitamin D seems to be a contributing factor in optimizing cardiac function [29] (Figure 2). First, in patients affected with CHF, vitamin D deficiency has a high prevalence; it may activate the renin-angiotensin-aldosterone system, favor chronic inflammation, and cause endothelial damage that is implicated as contributing to hypertension, diabetes, and obesity [30]. By definition, patients with advanced HF and congestive signs have reduced mobility, exposure to sunlight, and physical activity, with a consequent alteration in bone and vitamin D metabolism [31]. A reduced absorption of nutrients due to intestinal congestion is relevant, and the use of diuretics and hyperaldosteronism can also play a role, with relevant losses of Ca and Mg, as well as the frequent association with diabetes and variable severity of concomitant renal insufficiency. The increased risk of osteoporosis and fractures is clear in such group of patients, but the restoration of adequate bone and muscle metabolism is paramount for the general improvement of patients with cardiac surgery, CHF, heart transplantation, and LVAD placement [32]. Wu et al. have highlighted that patients with HF have a high prevalence of severe bone remodeling (namely resorption), which has a link to mortality [33].

Guidelines on Vitamin D defined 50 nmol/L as “vitamin D requirement of nearly all normal healthy persons,” using bone health as the principal basis [34,35]. Similarly, critically ill patients display a very high prevalence of vitamin D deficiency, clearly associated with greater illness severity, morbidity, and mortality in both adult and pediatric intensive care unit (ICU) patients, as well as medical and surgical intensive care units [36]. In LVAD carriers, the concepts of metabolic support verified in critically ill and CHF patients fit very well. Also in this setting, it should still be determined whether low vitamin D is an innocent bystander, simply reflecting greater disease severity, or represents an independent and modifiable risk factor amenable to normalization through supplementation [37,38]. The question is meaningful since in LVAD implants many factors contribute to low levels: hemodilution, frequent renal failure, reduced production and conversion by the liver, altered mineral metabolism, reduced synthesis of vitamin D binding protein, higher consumption during the acute phase of disease and systemic inflammation, and increased tissue demand and enhanced catabolism of metabolites [39]. More data are emerging from basic science about immediate and late effects of vitamin D supplementation on endocrine, autocrine and paracrine, and genomic targets; it is likely that the mere monitoring of vitamin D supplementation based on bone turnover markers is not adequate for this purpose [40]. Moreover, in a setting of chronic patients, with a number of repeated confounding factors and concomitant risk factors, the use of mortality or the number of mechanical circulatory support as clinical outcomes, should be considered with caution when the effect of the vitamin D supplementation is tested [40]. However, differently from HF patients, LVAD was able to determine a transient effect of the FGF-23 (effector of vitamin D metabolism, but also linked to physical activity and kidney function), and to increase significantly the level of circulating vitamin D [39].

A specific effect of vitamin D on the immunity and prevention (and possibly also treatment) of infections has been advocated, and that may be critical for CHF patients with VAD, and when they proceed to heart transplantation [36,41]. Given the inflammation and immunosuppression in the setting of transplantation, any adjuvant treatment should be considered to reduce the impact of infections in the early VAD placement or transplantation for the single patient, and from a wide healthcare system view, since such patients when they experience an infection it is often a complicated one and may impair resource availability and the clinical outcome of the patient [42,43,44].

### 3.2. Considerations after LVAD Implant and Potential Implications for Supplementation

In intensive care, usually loading dose (followed by a daily dose) is necessary to improve vitamin D levels rapidly [45]. There is no consensus on the optimal level for vitamin D supplementation, ranging from 400 to 2000 IU daily [46]. A safe and commonly available dose of 25 μg vitamin D3 (1000 IU) raises 25-hydroxyvitamin D (25(OH)D) serum level by 15–25 nmol/L on average (over weeks/months) [46,47,48,49]. The toxicity is also variable; in fact, the upper daily limit suggested by the Endocrine Society is 10,000 IU [34], while the Institute of Medicine (USA) and The European Food and Safety Authority recommend staying below 4000 IU/day (100 µg) [50,51]. In any event, in clinical practice rarely are toxic levels of vitamin D reached, and the patients remain, most of the time, vitamin D deficient. Moreover, the availability of vitamin D supplementation still varies from hospital to hospital, and the two most frequent molecules available are cholecalciferol (needs conversion by the liver enzymes) and calcifediol (bypasses liver activity but still needs last activation). Awaiting specific evidence it is reasonable to adopt the already available recommendations for supplementation in critically ill patients according to the recent nutrition guidelines released by ESPEN.

## 4. Erythropoiesis-Stimulating Agents and Iron Supplementation

Numerous mechanisms beyond low cardiac output sustain impaired exercise tolerance in patients with chronic heart failure. Among them, anemia is well recognized [52,53,54]. In patients with HFrEF, anemia has multiple effects: on the one hand, it increases myocardial work in the attempt to augment oxygen delivery; on the other, increasing the Hb level with erythropoiesis stimulating agents (ESAs) decreases left ventricular ejection fraction (LVEF) and cardiac output, probably because of increased blood viscosity and decreased nitric oxide availability [55,56,57].

Anemia is very common in patients with heart failure (HF), affecting 50% of patients with acute decompensated HF [58,59,60]. Moreover, it is associated with mortality with a cut-off value of 12.0 g per deciliter [61]. Therefore, the hemoglobin level might simply be a marker of poor prognosis in heart failure rather than a therapeutic target.

The pathogenesis of anemia in HF is multifactorial, but iron deficiency (ID) is extremely common [54], with a reported prevalence of between 30 and 70% [61,62]. Patients with severe anemia often have features of worse HF, with more extensive left ventricular (LV) remodeling and higher levels of biomarkers of advanced HF, higher inflammatory and collagen markers, and worse renal function. These factors lead to a picture of anemia of chronic disease, with defective iron utilization, inappropriate erythropoietin responsiveness, and depressed bone marrow function.

The criteria for diagnosing absolute iron deficiency is a serum ferritin level <100 ng/mL, and the criteria for functional iron deficiency is a ferritin serum level between 100 and 300 ng/mL, combined with a transferrin saturation <20%.

Small studies have shown that erythropoiesis-stimulating agents (ESAs) improve subjective measures of HF. However, a large pivotal outcome trial found that the ESA darbepoetin alfa did not improve long-term outcomes in patients with HF with reduced ejection fraction, and instead was associated with adverse effects. Studies using IV iron showed better results, with improvements in subjective and objective end points.

### 4.1. Targeting Iron Deficiency and Anemia in Heart Failure

Oral iron supplementation is the standard therapy for patients with ID because it is convenient, readily available, and inexpensive.

However, in patients with HF, apart from gastrointestinal intolerance, oral iron is poorly absorbed because of elevated hepcidin, which inhibits iron absorption by reducing transmembrane ferroportin on enterocytes, thereby preventing iron transfer from enterocytes to the blood [62].

With this background, it is unlikely that co-administration of vitamin C will increase gastrointestinal iron absorption.

Treating iron deficiency in HF with intravenous iron administration results in a reduction of HF-related hospitalizations and severe adverse events, and improves HF symptoms and quality of life [53,54]. Treatment with intravenous ferric carboxymaltose in patients with chronic heart failure and iron deficiency, with or without anemia, improves symptoms, functional capacity, and quality of life [63]. This prompted the ESC HF guidelines to recommend (II a) intravenous iron administration be considered in symptomatic HF with reduced ejection fraction patients [64].

Gastrointestinal malabsorption, long-term aspirin use, and uremic gastritis may also precipitate iron-deficiency anemia. Though erythropoietin levels are elevated and correlate with disease severity in heart failure, the elevation is inadequate.

Small studies have suggested that increase in hemoglobin with an erythropoiesis-stimulating agent (ESA) may improve functional capacity and reduce hospitalization in patients with heart failure and anemia [57]. Indeed, ESAs have not improved cardiovascular outcomes in anemic patients with chronic kidney disease, on the contrary it increased the risk of thrombotic events.

Swedberg et al. evaluated the effect of correcting anemia in patients with systolic heart failure with darbepoetin alfa [65], which led to an increase in the hemoglobin level. Despite this improvement, the darbepoetin alfa did not reduce the risk of the primary outcome of death or hospitalization for worsening heart failure; however, more patients had fatal or nonfatal strokes in the darbepoetin alfa group than in the placebo group. Fatal or nonfatal stroke occurred in 3.7% in the study group, and 2.7% in the placebo group (*p* = 0.23). In light of their findings, darbepoetin alfa is not recommended in this setting.

Similarly, the Trial to Reduce Cardiovascular Events with Aranesp Therapy (TREAT) [66], showed a significant increase in thromboembolic events in patients receiving darbepoetin alfa.

### 4.2. Considerations after LVAD Implant and Potential Implications for Supplementation

**Figure 3:**
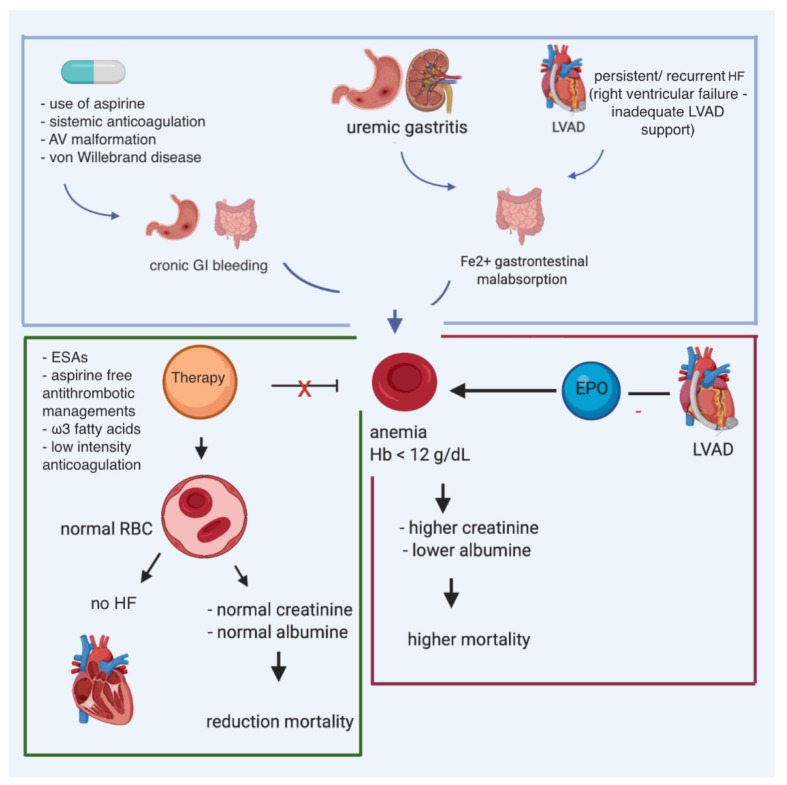
Multiple causes of anemia in patients affected with heart failure and carrying LVAD, and potential impact of iron supplementation and erythropoietin.

Data on ID are lacking in patients who have transitioned toward LVAD. Despite the evident harmful effects of ID, and the positive results of intravenous iron administration in chronic HF patients, data in LVAD patients is limited. Gastrointestinal malabsorption due to right ventricular failure, long-term aspirin use, and uremic gastritis may also contribute to iron-deficiency anemia. Indeed, LVAD patients are at an increased risk of gastrointestinal blood loss: the vicious circle of iron deficiency can worsen the right ventricle function, increasing the risk of right ventricular failure (Figure 3). Potentially, by early diagnosis and treating iron deficiency in LVAD patients, the right ventricle function might be optimized, preventing severe right ventricular failure, and improving patient outcomes. However, this should be further investigated.

Vrtovic et al. reviewed the data of 65 consecutive patients who underwent LVAD support for at least 6 months. Anemia, defined as hemoglobin levels <12 g/dL, was present in 30/65 patients (46%) after 6 months of LVAD support. Anemic patients had higher levels of pre-implant creatinine (1.8 + 0.8 vs. 1.4 + 0.5 mg/dL; *p* = 0.04). The presence of anemia after 6 months correlated with higher levels of creatinine and blood urea nitrogen and lower levels of albumin. Multivariate Cox proportional hazards regression analysis revealed that levels of hemoglobin, creatinine, and albumin were associated with all-cause mortality at 15 months. Long-term survival was two times higher in non-anemic patients after 6 months of LVAD support than in anemic patients (*p* = 0.01) [67].

Use of ESAs in patients with LVADs may minimize blood transfusions and decrease allosensitization. The inflammatory milieu that ensues during LVAD support can suppress erythropoiesis and diminish its effectiveness. Anemic LVAD patients have lower-than-expected circulating erythropoietin levels. ESAs are interesting for patients listed for heart transplant, as reducing transfusions and subsequent risk of developing anti-HLA antibodies might delay procurement of an appropriate donor. Post-transplant, allosensitization can also lead to higher rates of organ rejection and allograft vasculopathy.

However, these potential benefits of ESAs in LVAD-supported patients should be compounded with their potential risks of thrombotic complications, which is concerning because LVADs are sensitive to pump thrombosis (PT). In a study by Nassif et al. [68] ESA use in patients with the HeartMate II had higher rates of suspected PT (hazard ratio (HR): 2.35; 95% confidence interval (CI): 1.38 to 4.00; *p* = 0.002). For every 100-unit increase in cumulative ESA dosage, the hazard of suspected PT increased by 10% (HR: 1.10; 95% CI: 1.04 to 1.16; *p* < 0.001).

Mechanisms for development of pump thrombosis in patients receiving ESAs are likely multifactorial, and do not necessarily involve hyperviscosity.

On the basis of the currently available evidence, we envision a role for ESAs in the following scenarios:

- Preoperative anemic patients who are on IV anticoagulants, with the target of perioperative reduction of PRBC transfusions.
- Postoperative use started only when therapeutic anticoagulation is reached.
- During LVAD support in patients who require transfusions, and are still symptomatic, with renal failure and/or RVF.

Care should be taken according to the pump type: more liberal use might be justified with the HeartMate III in light of the lower rate of pump thrombosis.

The risk/benefit profile of ESA in anemic patients with GI bleeding and managed with low dose anticoagulation has yet to be determined.

## 5. The Role of Testosterone in Chronic Heart Failure and after VAD Implants

The relationship between androgens and cardiovascular disease has been studied for many decades, and yet there are still many poorly understood aspects [69]. Testosterone (TT) is produced in the Leydig cells in men under the control of luteinizing hormone, which is in turn controlled by gonadotropin-releasing hormone (GnRH). The important precursors to TT are dehydroepiandrosterone sulphate (DHEAS) and androstenedione. While the majority of the TT secreted in the blood is bound to proteins such as sex-hormone-binding globulin (SHBG), the free testosterone is the hormone that is transported within the cell and converted to dihydrotestosterone (DHT) by 5-Alpha reductase. TT and DHT both act on the androgen receptor (AR), which is responsible for the direct genomic effects by affecting transcription within the nucleus [70]. In addition, there appears to be a faster second pathway of action via a SHBG that attaches to a SHBG receptor to form a complex leading to the activation of cAMP and protein kinase-A. This pathway via SHBG plays a bi-directional role in testosterone regulation. Not only does it increase the affinity of AR to testosterone, but is also affected by testosterone, which can modify its action at different target cells [71,72]. The main source of androgens in women, in contrast, is the adrenal medulla, and the levels are considerably lower.

### 5.1. Effects on Cardiac Function

In addition to their well-known anabolic effects, androgens play an important role in the vascular system by supporting endothelial cell growth and proliferation, likely via vascular endothelial growth factor (VEGF)-mediated mechanism, and assisting in repair of injured endothelium by stimulation the endothelial progenitor cells [73]. Likely the most important effect of androgens is their effect on vascular tone [74]. It has shown to lead to relaxation of the vascular smooth muscle cells via multiple pathways, the most important being NO generation [75].

**Figure 4:**
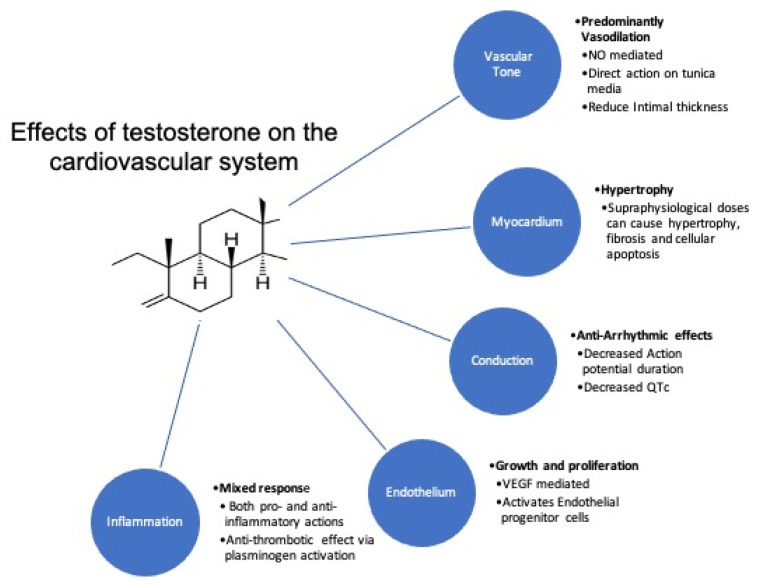
Summary of the effects of testosterone on cardiovascular system. Abbreviations: NO, nitric oxide; VEGF, vasoactive endothelial growth factor; QTc, corrected Q-T interval.

Androgens could also potentially play a role in myocardial growth and function. TT is the most potent anabolic hormone, which affects muscle growth likely by regulating the levels of IGF-1 and protein-3 [76,77]. Though there are no studies detailing their direct effects on the myocardium, a surrogate has been used to study patients who have abused anabolic steroids, in whom it causes cardiac hypertrophy and subsequent cardiomyopathy [78]. The summary of actions of androgens on the cardiovascular system is shown in Figure 4.

Blood androgen levels have been intrinsically related to cardiovascular health. Studies have shown an association between HF and a high prevalence of decreased total TT, free TT, and DHEAS, as well as other anabolic hormone like IGF-1, and an increase in the levels of SHBG [79,80]. The proportion of men who have low free TT levels among men with heart failure has been estimated to be anywhere between 21% and 65% [79,81,82]. It may very well be that low levels serve as a marker for a generalized catabolic state due to the HF, which activates neuroendocrine and inflammatory pathways associated similarly with other endocrine dysfunction [83].

Androgen levels also seem to be a predictor of outcomes. Low levels are associated with an increased risk of morbidity, as well as all-cause mortality and cardiovascular death among the general population. Recently the Atherosclerosis Risk in Communities (ARIC) study was published, which specifically studied the risk for heart failure in both men and women, and showed that lower levels of endogenous testosterone in men, DHEA-sulphate in men, and post-menopausal women were associated with subsequent development of HF [84,85,86,87,88]. Within the group of patients with HF, Jankowska et al. and Wehr et al. showed that, indeed, below normal androgen levels in HF patients were an independent predictor of mortality [79,89].

Unfortunately, androgen replacement in those patients with heart failure and low endogenous testosterone levels has not been shown to improve the LVEF in both men and women, but appears to improve exercise capacity [90,91,92,93,94].

### 5.2. Considerations after LVAD Implant and Potential Implications for Supplementation

The association of benefits and risk of androgens in patients with heart failure is expected to continue with patients with LVADs, though there has been limited study in this subset of the population.

Simsek et al. recently published a study examining FT and TT levels in patients receiving LVAD implantation [82]. They showed that the preoperative prevalence of low level of endogenous FT and TT among patients receiving LVAD support was higher than in the general HF population.

In addition, they reported that low FT and TT levels were predictors of mortality after a 4-year follow-up period. As pointed out by the authors themselves, their study does not take into consideration any changes to the testosterone levels after implantation or any effects attributable to that. It is likely that there may be a degree of correction of androgen levels after LVAD implantation, as one preliminary report shows, which is related to the improvement in metabolic dysfunction, which could lead to a survival benefit [6].

Another aspect to consider is patients’ functional capacity post-implantation. Patients usually have some exercise capacity recovery after LVAD implantation [95]. Given the literature showing the benefit of testosterone replacement in improving the exercise capacity in patients with HF, we propose that there may also be a similar effect on patients after LVAD implantation.

While there is no consensus or published guidelines, based on the current evidence it may be acceptable to consider replacing a diagnosed deficiency in endogenous androgen levels in patients with HF both pre- and post-LVAD implantation.

## 6. Effect of Estrogens and Progesterone on Cardiovascular Disease

The effects of estrogen in heart failure continue to be poorly modeled, though there is consensus that 17-Beta-estradiol is cardioprotective. While paracrine estrogen secretion from tissues like adipose, bone, vascular bed via aromatization may be responsible for its local action, it does not play a significant role until menopause, when ovarian secretion drastically reduces [96]. Estrogens actions via an estrogen receptor (ER) that exerts a slower genomic effect in the nucleus via co-ordination with E2 responsive elements (ERE), and also a faster P13K/AKT pathway which activates NO release [97].

Newer evidence seems to suggest that progesterone, which is primarily secreted in the corpus luteum of the ovary also has cardiovascular protective effects via a myriad of mechanisms. It also exhibits similar genomic effects via the nuclear progesterone receptors, as well as the non-genomic membrane progesterone receptor via NO synthesis in the endothelial cells [98].

The biochemical process by which these hormones exert these effects on the cardiovascular system has been hypothesized to be via an increase in angiogenesis via VEGF, decrease in reactive oxygen species production, reduction of fibrosis by reducing fibroblast proliferation, migration, and decreased collagen deposition [99].

### Hormonal Replacement Therapy

Though laboratory studies suggest cardio-protection from estrogens and progesterone, that effect does not translate into clinical benefit when hormonal replacement therapy (HRT) is considered. The Heart and Estrogen/progestin Replacement Study (HERS) as well as the Women’s Health Initiative (WHI) both showed no benefit in HRT. These findings are again controversial and there has been wide review of both these studies. More recent studies have noted that when HRT was initiated in relatively younger women and at an earlier stage of menopause, it reduced the total mortality and cardiovascular events; but had no effect or a possible adverse effect when started in older post-menopausal women. These findings have been suggested to explain a “menopausal transition” that leads to cardiovascular changes and fibrosis during this period, and shines the focus on the timing of initiation of HRT [100].

Based on the current evidence, initiation or continuation of HRT in the perioperative setting for women having LVAD implantation should be based on non-cardiovascular indications. The American College of Cardiology (ACC) and American College of Obstetrics and Gynecology (ACOG) currently do not recommend the use of HRT for primary or secondary prevention with respect to cardiovascular disease, including patients with heart failure since they have not shown any benefit [101]. Another consideration is the risk of deep vein thrombosis in women on HRT with risk factors, though this risk is somewhat mitigated by the fact that patients with LVADs are systematically anti-coagulated. One case for the use of HRT in patients with LVADs is the disproportionate outcomes of strokes after LVAD implantation among women [102]. Though the INTERMACs report studied a smaller ratio of women, it showed a higher risk of both ischemic as well as non-ischemic stroke in post-menopausal women with LVADs.

While at present there are very limited data to make robust recommendations on the use of HRT in women with LVADs, it is likely that peri- menopausal women, who are on anticoagulation with an LVAD may benefit from HRT, without the risks previously ascribed to it. There is still a need for more robust clinical trials carried out among this population, and future studies will help shed more light on this area.
